# Factors Associated With Viral Load Suppression Among Adolescents and Young Adults Living With HIV on ART in Rwanda: Data From Rwanda HIV Case‐Based Surveillance, 2019–2025

**DOI:** 10.1155/jotm/5304043

**Published:** 2026-08-25

**Authors:** Gallican N. Rwibasira, Jean Claude Kwizera, Steven Karera, Tafadzwa Dzinamarira, Charles Holmes, Ayman Ahmed, Albert Tuyishime, Eric Remera, Daniel Henry Paris, Tracy R. Glass

**Affiliations:** ^1^ Rwanda Biomedical Centre, Kigali, Rwanda, rbc.gov.rw; ^2^ Swiss Tropical and Public Health Institute, Allschwil, Basel, Switzerland, swisstph.ch; ^3^ University of Basel, Basel, Switzerland, unibas.ch; ^4^ ICAP Global Health, Kigali, Rwanda; ^5^ School of Health Systems and Public Health, University of Pretoria, Pretoria, South Africa, up.ac.za; ^6^ Center for Innovation in Global Health, Georgetown University, Washington, DC, USA, georgetown.edu; ^7^ Institute of Endemic Diseases, University of Khartoum, Khartoum, Sudan, uofk.edu; ^8^ Pan-African One Health Institute (PAOHI), Kigali, Rwanda

**Keywords:** adolescents and young adults, case-based surveillance, HIV, viral load suppression

## Abstract

**Background:**

Adolescents and young adults living with HIV (AYALHIV) in sub‐Saharan Africa have lower viral load (VL) suppression (VLS) rates than older adults. Rwanda lacks national‐level evidence on predictors of VLS in this age group. This study assessed demographic and clinical determinants of VLS among AYALHIV aged 15–24 years on antiretroviral therapy (ART), using national case‐based surveillance (CBS) data.

**Methods:**

We conducted a retrospective cohort analysis of AYALHIV enrolled in Rwanda’s CBS system who initiated ART between January 2019 and December 2025 and had a documented VL at least six months after ART initiation. The primary outcome, VLS, was defined as VL ≤ 1000 copies/mL. Modified Poisson regression with robust, facility‐clustered standard errors was used to estimate prevalence ratios (PRs); missing covariate data were addressed using multiple imputation (*m* = 20), pooled using Rubin’s rules.

**Results:**

A total of 4140 participants across 458 health facilities were included. Most were female (88%) and aged 20–24 years (90.7%); 91.6% were initiated on a dolutegravir‐based regimen, 98.7% demonstrated good adherence, and overall, 96.1% achieved VLS. In multivariable analysis, good adherence was the only factor independently associated with VLS, corresponding to a 70% higher prevalence of suppression relative to bad/moderate adherence (adjusted PR = 1.70; 95% CI: 1.33–2.19; *p* < 0.001).

**Conclusion:**

VLS among AYALHIV aged 15–24 years engaged in Rwanda’s HIV care program is high, exceeding the UNAIDS 95% target. Country HIV programs should prioritize adherence‐focused interventions and proactively address underlying barriers to adherence in order to sustain optimal treatment outcomes in this population.

## 1. Background

Over the past 2 decades, the widespread scale‐up of antiretroviral therapy (ART) has contributed greatly to progress in the global HIV response [1]. According to the Joint United Nations Programme on HIV/AIDS (UNAIDS) Global Update, the trend of new HIV infections declined by 39%, and AIDS‐related deaths dropped by around 50% in 2023 compared to 2010. The same report also showed that 86% of people living with HIV (PLHIV) knew their status, 89% of those were enrolled on treatment, and 93% achieved viral load suppression (VLS) across all ages [2]. However, adolescents and young adults living with HIV (AYALHIV) aged 15–24 lagged behind, with approximately 61% being aware of their status, 84% on ART, and only 77% virally suppressed [3].

The World Health Organization (WHO) recommends viral load (VL) testing as a gold standard for monitoring the effectiveness of ART. WHO defines VLS as a VL with HIV RNA ≤ 1000 copies/mL. The main outcome of ART success is a sustained VLS after 6 months of ART initiation. According to the WHO, virological failure is indicated by two consecutive VL > 1000 copies/mL after six months of ART [4]. VLS outcomes are influenced by multiple factors, including patient‐level determinants (such as adherence, age, and sex), clinical factors (like baseline CD4 count or advanced HIV disease), regimen‐related aspects, and socio‐environmental influences such as socioeconomic status and stigma [5, 6]. Despite global attention and efforts toward the HIV response, VLS rates among AYALHIV aged 15–24 years, who represent around 8% of all PLHIV globally [3], remain heterogeneous.

In sub‐Saharan Africa (SSA), several studies have identified critical predictors of unsuppressed VL. A recent systematic review covering over 750,000 individuals found that children and adolescents had the highest rate of viral nonsuppression (28%), with risk factors including male sex, older adolescent age, adherence lapses, lower CD4 counts, regimen switches, longer time on ART, and socio‐economic deprivation [7]. Country‐specific studies, such as those from Kenya, Tanzania, Uganda, and South Africa, corroborate these trends [8–10]. For instance, in Homa Bay County, Kenya, adolescents with good adherence to ART and CD4 counts above 500 cells/mm^3^ were significantly more likely to achieve VLS [11], and in Tanzania, not taking a dolutegravir (DTG)‐based regimen was found to be associated with nonsuppressed VL [10]. These findings suggest similar multifactorial drivers of viral outcomes across the SSA Region.

The unsuppressed VL rates reported in a systematic review and meta‐analysis conducted between 2010 and 2024 among children and adolescents were around 26.5% and 24.7% among adolescents aged 10–19 years [12]. Another meta‐analysis of 15 studies in SSA found an overall VLS rate of 55% among adolescents, with significantly lower rates observed in the presence of social and therapy‐related barriers [13]. Existing evidence shows the geographical disparities in VLS rate across different countries of SSA; for example, in Nigeria, a study showed VLS varying from 55.6% to 64.0% [14]; in Eswatini, a cohort of 911 adolescents on ART showed VLS at 88.5% [15], while a study in South Africa’s Free State province showed VLS at 78% [16].

In Rwanda, the 2018–2019 Rwanda Population HIV Impact Assessment (RPHIA) survey reported that although 90.1% of adults on ART achieved viral suppression, the VLS rate among adolescents aged 10–19 years was notably lower, at 86% [17]. A 2018 study from 10 health centers in Rwanda demonstrated a 91% suppression among individuals ≥ 15 years retained on ART, but those aged 15–24 were less likely to be suppressed compared to those ≥ 50 years [18].

To date, Rwanda lacks national‐level studies that comprehensively assess predictors of VLS among AYALHIV aged 15–24 years. This particular group represents a uniquely vulnerable population in the global HIV response, often experiencing poorer outcomes across the treatment cascade. Studies have consistently shown that this age group has lower rates of ART adherence and VLS compared to older adults [19, 20]. The challenges contributing to poor adherence and nonsuppression among youth include psychosocial factors such as stigma, lack of disclosure, limited family or peer support, and mental health issues, as well as structural barriers like weak transition systems from pediatric to adult care and reduced engagement with health services [21, 22]. There is increasingly growing evidence across SSA that adolescents had the highest viral nonsuppression rates, with adherence lapses being a major determinant [23]. These disparities underline the critical need for age‐specific data to inform tailored interventions.

In Rwanda, while national‐level HIV data systems like case‐based surveillance (CBS) have matured, there remains a gap in data specifically targeting AYALHIV. Unlike routinely aggregated HIV program reports, Rwanda’s CBS system captures individual‐level, longitudinally updated clinical data linked to each client across all ART‐providing facilities nationwide [24], making it uniquely suited to examine person‐level predictors of VLS, including adherence and clinical history, that cannot be assessed from routine aggregate reporting systems. This study, therefore, focused on analyzing CBS data among individuals aged 15–24 years who initiated ART from January 2019 to December 2025, to understand the demographic and clinical predictors of VLS in this predefined underserved and high‐risk population category.

## 2. Methods

### 2.1. Study Design

This study used a retrospective cohort design, in which routinely collected programmatic surveillance data were analyzed to assess demographic and clinical predictors of VLS among AYALHIV who initiated ART in Rwanda. Within this retrospective cohort, VLS was assessed cross‐sectionally, using each participant’s first VL result recorded at least 6 months after ART initiation, as a programmatic indicator of the primary ART‐effectiveness endpoint.

### 2.2. Study Setting and Population

The study was conducted in Rwanda, where, as of December 2025, a total of 232,113 PLHIV were receiving ART countrywide, including 15,572 AYALHIV aged 15–24 years (HMIS aggregate data). The CBS system was launched in 2018, initially as a pilot intervention in 23 facilities in the City of Kigali to enhance active case finding (ACF) and partner notification services. Following complete scale‐up in 2019, the system was extended to all 589 ART‐providing health facilities nationwide. CBS was designed to strengthen HIV case detection by identifying individuals who test positive (index cases) and offering them counseling to support notification of their potential partners, including sexual partners, family members, and other relevant social network contacts. This comprehensive surveillance approach involves longitudinal follow‐up of PLHIV on ART to closely monitor their clinical outcomes and HIV transmission risk.

Although the CBS system enrolls PLHIV aged 15 years and older, this analysis was restricted to a narrower subset in line with the study objective. Participants were included if they were aged 15–24 years, enrolled in the CBS system, had initiated ART between January 2019 and December 2025, and had at least one documented VL result recorded after six months on ART. Participants were excluded if they had been on ART for less than 6 months at database closure (and were therefore not yet eligible for VL testing under national guidelines) or if they had been on ART for 6 months or more but had no VL result recorded in the CBS system, regardless of the reason for the missing result; this second group was accordingly excluded from the analytic sample.

### 2.3. Data Collection Procedures and Quality Safeguard

Data for this analysis were extracted from Rwanda’s national CBS database, which is integrated into the District Health Information Software 2 (DHIS2) platform and covers all national health facilities implementing the CBS system, for the period January 2019 to December 2025. Data are captured and stored at the individual (client) level within DHIS2, rather than in aggregated form, allowing demographic, clinical, behavioral, and outcome information to be linked longitudinally for each client over time.

**FIGURE 1:**
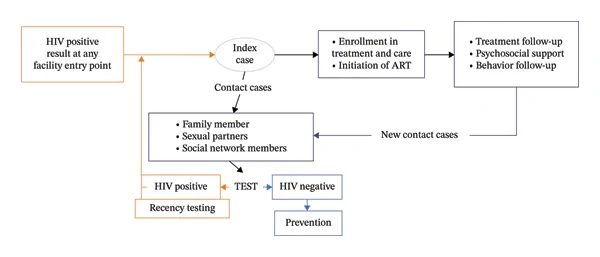
HIV case‐based surveillance structure in Rwanda.

The CBS system in Rwanda operates through two main components: ACF and routine case surveillance, also referred to as longitudinal follow‐up (Figure 1). The ACF component focuses on proactively identifying new HIV cases within the community, whereas the routine surveillance arm involves ongoing monitoring of individuals diagnosed with HIV who are on ART, assessing treatment adherence, treatment outcomes including VLS, and socialdemographic parameter changes.

In practice, to be enrolled in CBS, individuals aged 15 years and above newly diagnosed with HIV or already on ART are counseled and provide verbal consent before enrollment. To safeguard data quality, a trained health care provider completes a confidential case report form (CRF) for each client, capturing demographic, clinical, behavioral, and outcome data, including VL results and treatment status. This standardized workflow, overseen by RBC, requires the CRF to be forwarded to a facility‐level trained data manager, who reviews it for completeness and internal validity, resolving any missing or inconsistent entries with the provider, before the record is entered into DHIS2 under the client’s unique patient identifier (UPID); the paper CRF is then archived for reference. As a client’s status evolves, the provider updates the relevant CRF sections at each follow‐up visit, with corresponding updates entered into DHIS2 under the same UPID, enabling longitudinal linkage of each client’s records over time. At the central level, RBC conducts routine (quarterly) analysis of reported CBS data, with results and feedback disseminated back to facilities to support continuous data quality improvement.

### 2.4. Ethical Consideration

The CBS system was launched jointly by the Rwanda Biomedical Centre (RBC) and the U.S. Centers for Disease Control and Prevention (CDC). It was approved in 2018 (tracking number 2018–309) and subsequently reviewed by the Rwanda National Ethics Committee (RNEC)—approval No. 907/RNEC/2019, November 18, 2019—which approved verbal, rather than written, informed consent for active CBS, recency testing, and routine follow‐up, in line with national HIV treatment program procedures. It was granted a non‐research determination (NRD) in 2021 by the RBC and CDC, after which it became part of Rwanda’s routine surveillance system to strengthen ACF. All data included in this study were generated through this programmatic national surveillance system, without personal identifiers being shared with third parties. Access to and extraction of the de‐identified CBS dataset for this secondary analysis was administratively authorized by the RBC under its institutional data‐sharing governance procedures.

### 2.5. Study Variables

#### 2.5.1. Outcome Variable

This analysis used each participant’s first recorded VL result in the CBS database. The primary outcome, VLS, was defined per WHO guidelines as VL ≤ 1000 copies/mL, with VL > 1000 copies/mL classified as unsuppressed [4]. This threshold was applied uniformly to all participants regardless of ART regimen—DTG or non‐DTG–based—and was assessed using a single first VL measurement obtained at least 6 months after ART initiation.

The programmatic outcome variables included patient outcome status defined at database closure as alive and on ART when patients were actively receiving ART and documented as in care; lost to follow‐up when patients had not attended a scheduled ART refill or clinical appointment and were unreachable for at least 90 days after the last expected visit; and death when patients were recorded as deceased in the surveillance system.

#### 2.5.2. Predictors

The sociodemographic variables were based on information reported in the CBS system and included sex (male, female), age group (15–19, 20–24), and marital status (single, never married), in union (married or cohabiting), or divorced/separated).

The clinical variables included baseline CD4 count (first documented CD4 measurement at or near ART initiation, categorized as < 200 cells/mm^3^, 200–499 cells/mm^3^, or ≥ 500 cells/mm^3^) [25] and WHO clinical stage at ART initiation (as per WHO staging guidelines I‐IV) [25]. The initial ART regimen was categorized as DTG‐based versus non‐DTG–based. ART regimens were defined as either first‐line or second‐line regimens, according to national guidelines at the time of VL measurement. Time on ART referred to the duration in months from ART initiation to the date of the first recorded VL test and was categorized as 6–11 months, 12–23 months, or ≥ 24 months. Adherence was assessed by the healthcare provider based on individual self‐report and/or pill count. The percentage adherence was calculated as the number of doses reportedly taken divided by the total number of doses prescribed during the reference period, multiplied by 100. PLHIV with ≥ 95% adherence were classified as good, those with 85%–94% as moderate, and those with < 85% as poor [26].

### 2.6. Statistical Analysis

Data were analyzed using R statistical software, Version 4.5.1 (R Foundation for Statistical Computing, Vienna, Austria, 2025). Descriptive statistics were calculated using frequencies and percentages for categorical variables and medians with interquartile ranges for continuous variables. Missing data were present for several covariates; these were assumed to be missing at random and addressed using multiple imputation by chained equations (*m* = 20 imputed datasets, mice package), with predictive mean matching used for continuous variables and logistic or multinomial logistic regression used for binary and multicategory variables, respectively. The imputation model included all covariates and the outcome variable.

Because the primary outcome (VLS) was common, occurring in over 90% of participants, modified Poisson regression with a log link was used to estimate prevalence ratios (PRs) rather than odds ratios to avoid overestimation of association measures inherent to logistic regression under high outcome prevalence. Univariate Poisson regression models with robust (Huber‐White, HC0) standard errors were first fitted to assess the unadjusted association between each independent variable and VLS. All predictor variables, selected a priori based on clinical relevance and prior literature, were then entered into a multivariable Poisson regression model. Multicollinearity among included predictors was evaluated using variance inflation factors (VIF < 5 considered acceptable), and a prespecified interaction term (baseline CD4 count × WHO clinical stage) was tested using a Wald test; as it was not statistically significant, it was not retained in the final model.

To account for potential correlation among clients attending the same health facility, standard errors in the multivariable model were clustered at the facility level using a cluster‐robust sandwich estimator, with facility‐level clustering additionally assessed via the intraclass correlation coefficient (ICC) from a null random‐intercept model as a sensitivity check. Model fit was evaluated using the Hosmer–Lemeshow test, Tjur’s *R* ^2^, and Brier score from an analogous logistic regression model, given the absence of standard goodness‐of‐fit statistics for Poisson models, and influential observations were identified using Cook’s distance. Estimates from the multivariable model were pooled across imputed datasets using Rubin’s rules. Adjusted prevalence ratios (aPRs) with 95% confidence intervals (CIs) were reported, and a *p* value < 0.05 was considered statistically significant.

## 3. Results

### 3.1. Study Participant Selection

**FIGURE 2:**
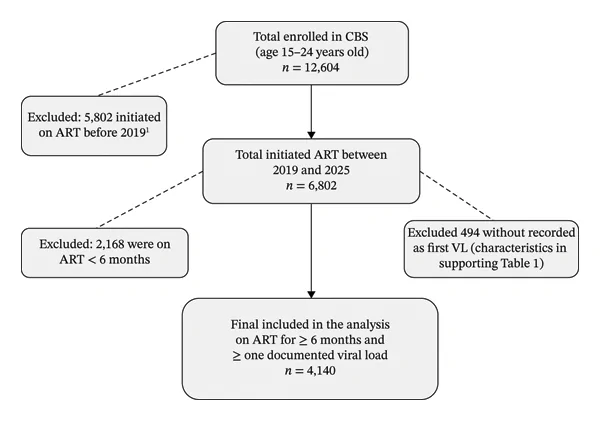
Study participant selection procedure and flow. ^1^Represent the number of AYALHIV who were initiated on ART anytime between 2004 and 2018 and were thereafter enrolled in the CBS system.

Of the 15,572 AYALHIV receiving ART as of December 2025, 12,604 (80.9%) were enrolled in the CBS system. Figure 2 presents the selection flow of the 4140 study participants included in the final analytic cohort. Compared with the 4140 participants included in the analysis, the 494 AYALHIV excluded for lack of a documented VL were somewhat younger (16% vs. 9.3% aged 15–19 years) and had a higher prevalence of baseline immunosuppression (CD4 < 200 cells/mm^3^: 12% vs. 7.9%; WHO stage III/IV: 2.1% vs. 1.2%), but were otherwise similar in sex, marital status, and initial ART regimen (Table S1).

### 3.2. Descriptive Characteristics of Study Participants

A total of 4140 AYALHIV‐aged 15–24 years living with HIV, enrolled on ART, and followed up through the CBS system between 2019 and 2025, were included in the analysis. Participants were drawn from 458 health facilities, with a median of 5 individuals per facility (IQR: 2–10). The majority were female (88%) and aged 20–24 years (90.7%), with a median age of 23 years (IQR: 21–24). At baseline, 92.1% had a CD4 count > 200 cells/mm^3^, and 98.8% were classified as WHO clinical stage I or II at ART initiation. Most participants (91.6%) were initiated on a DTG‐based regimen, 98.7% demonstrated good adherence prior to their first VL measurement, and overall, 96.1% had achieved VLS (≤ 1000 copies/mL) (Table 1).

**TABLE 1:** Baseline demographic and clinical characteristics of adolescents and young adults living with HIV on ART enrolled in the case‐based surveillance system in Rwanda.

Characteristics	*N*	Frequency (%)^∗^	Suppressed (%)^†^	Unsuppressed (%)^†^
Sex	4140			
Female		3633 (88%)	3497 (96.3%)	136 (3.7%)
Male		507 (12%)	482 (95.1%)	25 (4.9%)
Age group	4140			
15–19		386 (9.3%)	374 (96.9%)	12 (3.1%)
20–24		3754 (90.7%)	3605 (96.0%)	149 (4.0%)
Marital status	4133			
Single		2493 (60.3%)	2382 (95.5%)	111 (4.5%)
Married/cohabitant		1471 (35.6%)	1425 (96.9%)	46 (3.1%)
Divorced/separated		169 (4.1%)	165 (97.6%)	4 (2.4%)
Baseline CD4	3777			
< 200		297 (7.9%)	283 (95.3%)	14 (4.7%)
200–499		1664 (44.0%)	1607 (96.6%)	57 (3.4%)
500+		1816 (48.1%)	1741 (95.9%)	75 (4.1%)
WHO stage	4138			
I/II		4088 (98.8%)	3931 (96.2%)	157 (3.8%)
III/IV		50 (1.2%)	46 (92%)	4 (8.0%)
Initial regimen	4118			
DTG‐based		3774 (91.6%)	3630 (96.2%)	144 (3.8%)
Non‐DTG–based		344 (8.4%)	327 (95.1%)	17 (4.9%)
Time to VL test after ART initiation	4128			
6–11 months		2996 (73%)	2875 (96.0%)	121 (4.0%)
12–23 months		554 (13%)	533 (96.2%)	21 (3.8%)
24+ months		578 (14%)	559 (96.7%)	19 (3.3%)
Adherence	4027			
Bad/moderate		51 (1.3%)	33 (65%)	18 (35%)
Good		3976 (98.7%)	3874 (97.4%)	102 (2.6%)
Overall VL results	4140			
Suppressed (≤ 1000 cp/mL)		3979 (96.1%)	—	—
Unsuppressed (> 1000 cp/mL)		161 (3.9%)	—	—

*Note:* ART = antiretroviral therapy; CD4 = CD4+ T‐cell count; DTG = dolutegravir.

Abbreviations: VL = viral load, WHO = World Health Organization.

^∗^Column percentage.

^†^Row percentage.

Among the 4140 clients who were included in the analysis, 95.3% were alive and on ART, achieving high VLS (96.5%) with only 3.5% unsuppressed. In contrast, VLS rates for participants who were eventually lost to follow‐up or died were substantially lower (87.7% and 81.2%, respectively), indicating there were early signs of negative outcomes (Table 2).

**TABLE 2:** Descriptive comparison of VLS by participant’s treatment outcome (*N* = 4140).

	Client outcome		
	Alive and on ART	Stopped/loss to follow‐up	Death
Frequency (%)	3948 (95.3%)	176 (4.3%)	16 (0.4%)
Suppressed VL (%)	96.5%	87.7%	81.2%
Unsuppressed VL (%)	3.5%	12.3%	18.8%

*Note:* Presented descriptively; because outcome status was ascertained at database closure rather than at a fixed interval after the VL result, the temporal sequence between VL and outcome cannot be established, and this table should not be interpreted as showing a causal relationship.

### 3.3. Factors Associated With VLS

Table 3 presents univariate and multivariable modified Poisson regression models estimating PR for factors associated with VLS among AYALHIV on ART. Both models were fit on the full analytic sample (*N* = 4140) using multiple imputation (*m* = 20, pooled by Rubin’s rules) to account for missing covariate data, with facility‐clustered robust standard errors to account for within‐facility correlation. Good adherence was the only factor significantly associated with VLS in adjusted models, corresponding to a 70% higher prevalence of suppression relative to bad/moderate adherence (adjusted PR = 1.70, 95% CI: 1.33–2.19, *p* < 0.001). Sex, age group, marital status, baseline CD4 count, WHO clinical stage, initial ART regimen, and time to first VL test were not significantly associated with suppression after adjustment. No evidence of multicollinearity was observed (all VIFs < 2), model calibration was acceptable, and no influential observations materially affected the results (Tables S2, S4–S6).

**TABLE 3:** Univariate and multivariate modified Poisson regression of factors associated with VLS among adolescents and young adults living with HIV on ART enrolled in the case‐based surveillance system in Rwanda (*N* = 4140).

	VL category, *n* (row %)		Univariable (MI‐pooled)		Multivariable (MI‐pooled)	
	Suppressed	Unsuppressed	PR (95% CI)	*p* value	aPR (95% CI)	*p* value
Sex						
Female	3497 (96%)	136 (3.7%)	—		—	
Male	482 (95%)	25 (4.9%)	0.99 (0.97, 1.01)	0.234	0.99 (0.97, 1.01)	0.218
Age group						
15–19	374 (97%)	12 (3.1%)	—		—	
20–24	3605 (96%)	149 (4.0%)	0.99 (0.97, 1.01)	0.328	0.99 (0.97, 1.01)	0.152
Marital status						
Single	2382 (96%)	111 (4.5%)	—		—	
Married/cohabitant	1425 (97%)	46 (3.1%)	1.01 (1.00, 1.03)	0.042	1.01 (1.00, 1.02)	0.140
Divorced/separated	165 (98%)	4 (2.4%)	1.02 (0.99, 1.05)	0.125	1.01 (0.99, 1.04)	0.272
Baseline CD4						
< 200	283 (95%)	14 (4.7%)	—		—	
200–499	1607 (97%)	57 (3.4%)	1.01 (0.99, 1.04)	0.317	1.01 (0.98, 1.04)	0.547
500+	1741 (96%)	75 (4.1%)	1.01 (0.98, 1.04)	0.596	1.00 (0.97, 1.03)	0.962
WHO stage						
I/II	3931 (96%)	157 (3.8%)	—		—	
III/IV	46 (92%)	4 (8.0%)	0.96 (0.88, 1.04)	0.279	0.98 (0.91, 1.06)	0.634
Initial regimen						
DTG‐Based	3630 (96%)	144 (3.8%)	—		—	
Non‐DTG–Based	327 (95%)	17 (4.9%)	0.99 (0.97, 1.01)	0.340	0.98 (0.96, 1.01)	0.124
Time to VL test after ART initiation						
6–11 months	2875 (96%)	121 (4.0%)	—		—	
12–23 months	533 (96%)	21 (3.8%)	1.00 (0.98, 1.02)	0.785	1.00 (0.99, 1.02)	0.705
24+ months	559 (97%)	19 (3.3%)	1.01 (0.99, 1.02)	0.349	1.01 (1.00, 1.03)	0.070
Adherence						
Bad/moderate	33 (65%)	18 (35%)	—		—	
Good	3874 (97%)	102 (2.6%)	1.71 (1.33, 2.19)	< 0.001	1.70 (1.33, 2.19)	< 0.001

*Note:* — indicates the reference category. CD4 = CD4+ T‐cell count; WHO = World Health Organization (disease stage); DTG = dolutegravir. *N* = 4140 for all univariate and multivariable models (multiple imputation, *m* = 20, pooled using Rubin’s rules); facility‐clustered robust standard errors are used throughout.

Abbreviations: aPR = adjusted prevalence ratio, CI = confidence interval, MI = multiple imputation, PR = prevalence ratio.

## 4. Discussion

This national analysis of Rwanda’s CBS system found that 96.1% of AYALHIV aged 15–24 years who had a recorded first VL after at least six months on ART were virally suppressed (≤ 1000 copies/mL). In multivariable analysis, good adherence was the only factor independently associated with VLS, corresponding to a 70% higher prevalence of suppression relative to bad/moderate adherence. Sex, age group, marital status, baseline CD4 count, WHO clinical stage, initial ART regimen, and time since ART initiation were not independently associated with VLS after adjustment.

The point prevalence suppression rate observed here is high relative to pooled sub‐Saharan African estimates for this age group, where unsuppressed viremia among children and adolescents has been reported at 26%–28% in recent systematic reviews and meta‐analyses [7, 12]. It is also higher than the 86% suppression reported among 15–24‐year‐olds in the 2018–2019 RPHIA [17]. This difference is very likely attributable to differences in the populations captured: RPHIA sampled the general community, including individuals with HIV who were undiagnosed or not yet linked to care, whereas our analytic sample was restricted, by design, to AYALHIV already enrolled in CBS, initiated on ART, and retained long enough to have a documented VL. Our estimate is also relatively higher than the previous Rwandan facility‐ and program‐based cohort reporting suppression among PLHIV retained on ART under the Treat All strategy [18] and is consistent with programmatic reports of sustained high performance along Rwanda’s HIV care continuum more broadly [27–29].

Good adherence to ART was the only independently significant predictor of VLS in this cohort. This finding is consistent with prior evidence identifying adherence as the most proximal correlate of virologic outcomes among adolescents and young people, both globally [19] and across SSA [7, 13]. It also aligns with the well‐established biological mechanism whereby consistent adherence sustains adequate drug concentrations, thereby suppressing viral replication, limiting the emergence of drug‐resistant mutations, and enabling optimal treatment outcomes [30, 31]. This estimate should, however, be interpreted with some caution: Only 51 participants (1.3%) were classified as having bad/moderate adherence, and this small reference group limits the precision of the effect estimate despite its statistical significance. Yet, taken together, these findings reinforce adherence as the principal, modifiable lever for achieving and sustaining viral suppression. This underscores the need for programs serving AYALHIV to prioritize adherence‐focused counseling and to proactively address the underlying barriers to adherence, including stigma, nondisclosure of HIV status, limited family and peer support, and mental health challenges [21, 22].

Sociodemographic and clinical characteristics were not independently associated with VLS in this cohort. Notably, sex was not significantly associated with suppression despite the cohort being 88% female. This finding contrasts with a recent SSA systematic review, which reported that male sex was associated with poorer VLS in the adolescent population [12]. However, the proportion of VLS observed in our cohort (96% among females vs. 95% among males) is consistent with programmatic data from 21 PEPFAR‐supported SSA countries, which found viral suppression to be marginally, though statistically significantly, higher among women than men at the population level (93.0% vs. 92.0%) [32]. The pronounced female predominance in our sample relatively exceeds the sex distribution typically reported for this age group [27, 28] and likely reflects differential entry into HIV testing, care, and CBS enrollment—through antenatal and reproductive health platforms and services targeting adolescent girls and young women [26, 33]—rather than a true difference in treatment response once on ART and may not reflect the sex distribution of all AYALHIV in Rwanda.

Although 91.6% of participants were initiated on a DTG‐based regimen, consistent with Rwanda’s national scale‐up of DTG since 2018 [34], the initial regimen was not independently associated with VLS in the adjusted model. This is at variance with clinical trial and cohort evidence demonstrating virologic superiority of DTG‐based regimens over non‐DTG regimens in children, adolescents, and young adults elsewhere in SSA and globally [35–39], including a rural Tanzanian cohort in which DTG‐based treatment was strongly associated with suppression [40]. The absence of a significant association in our data most likely reflects a ceiling effect with suppression already at 96% overall and only 8.4% of participants on non‐DTG regimens; there may be little residual variability left for regimen type to explain. On the basis of this analysis alone, therefore, we cannot attribute Rwanda’s high population‐level VLS specifically to DTG rollout; this remains a plausible contributor supported by external evidence and by the temporal association between DTG scale‐up and rising national suppression rates [41], but it is not a finding directly demonstrated by our multivariable analysis.

The lower proportion of VLS observed among participants who were subsequently recorded as lost to follow‐up (87.7%) or who died (81.2%), compared with those alive and in care (96.5%), is a descriptive but clinically important pattern. Unsuppressed viremia and adverse downstream outcomes are known to be linked bidirectionally in HIV care—poor virologic control can precede disengagement and mortality, while disengagement can also delay or preclude subsequent testing and re‐suppression [42–45]—and because outcome status was ascertained at database closure rather than at a fixed interval after the index VL, the temporal sequence between the first VL result and the later outcome cannot be established with certainty in this analysis. We therefore interpret this finding as hypothesis‐generating: It reinforces that an unsuppressed first VL result may serve as an early, actionable signal warranting intensified follow‐up rather than as evidence of a causal pathway.

### 4.1. Study Strengths and Limitations

To our knowledge, this is the first analysis to use individual‐level national surveillance data to examine correlates of VLS specifically among AYALHIV in Rwanda. Drawing on 4140 participants across 458 facilities nationwide, the large sample size enhances the generalizability of our findings to the national AYALHIV population and to similar settings across SSA. Methodologically, the use of modified Poisson regression to estimate PRs, rather than odds ratios, avoids the well‐documented overestimation of effect size by logistic regression when an outcome is as common as VLS was in this cohort (96.1%). Facility‐clustered robust standard errors accounted for the non‐independence of observations within facilities, while multiple imputation across 20 datasets, pooled using Rubin’s rules, addressed missing data. Model diagnostics—including VIFs, the Hosmer–Lemeshow test, and Cook’s distance—further support the robustness of the reported adherence‐VLS association.

Several limitations should be acknowledged. First, the nature of this study design cannot establish causality, and the observed associations, including that between adherence and VLS, should be interpreted as such. Second, VLS was defined using each participant’s first recorded VL after at least 6 months on ART; this design cannot capture subsequent virologic rebound or re‐suppression and therefore does not reflect sustained VLS over time. Third, the exclusion of 494 participants with no documented VL may have introduced selection bias, potentially leading to an overestimation of VLS if those without documented results were, in fact, less adherent to ART. Fourth, several psychosocial and structural determinants known to influence both adherence and VLS in this age group—including disclosure status, stigma, mental health, schooling, and distance to facility—are not captured in the CBS dataset, so residual confounding of the adherence‐VLS association by these unmeasured factors cannot be excluded. Fifth, adherence was classified using provider‐assessed self‐report and/or pill count, measures that are prone to social desirability and recall bias and tend to overestimate true adherence; this likely explains why only a small proportion of participants (1.3%) were classified as having poor/moderate adherence, and such misclassification would typically bias the observed adherence–VLS association toward the null. Finally, generalizability is constrained to AYALHIV aged 15–24 years, as the Rwandan CBS does not capture individual‐level data for adolescents under 15 years; findings may therefore not generalize to younger adolescents or to those not currently on ART.

## 5. Conclusion

This study demonstrates that among adolescents and young adults aged 15–24 years living with HIV who were engaged in Rwanda’s HIV care program and had documented VL results, VLS is high (96.1%), exceeding the UNAIDS 95% target and remaining largely uniform across demographic and clinical categories. Good adherence was the only factor independently and significantly associated with suppression in adjusted analysis, identifying it as the principal modifiable target for programmatic action in this population. These findings should, however, be interpreted in light of the study’s design, which precludes causal inference, and its dependence on provider‐assessed adherence measures that are prone to overestimation. Findings are not generalizable beyond this group and may not extend to younger adolescents or to AYALHIV not yet linked to care or tested. Notwithstanding these limitations, this national analysis offers the first individual‐level evidence on correlates of VLS among AYALHIV in Rwanda and underscores the need for programs to strengthen adherence‐focused counseling and support in order to sustain optimal treatment outcomes in this population. Future research should prioritize extending surveillance to younger adolescents and evaluating the effectiveness of psychosocial and adherence‐support interventions on VLS specifically within this population in Rwanda.

**Nomenclature**
ACF: Active case finding
aPR: Adjusted prevalence ratio
ART: Antiretroviral therapy
AYALHIV: Adolescents and young adults living with HIV
CBS: Case‐based surveillance
CD4: CD4+ T‐cell count
CDC: U.S. Centers for Disease Control and Prevention
CI: Confidence interval
CRF: Case report form
DHIS2: District Health Information Software 2
DTG: Dolutegravir
HIV: Human immunodeficiency virus
ICC: Intraclass correlation coefficient
MI: Multiple imputation
NRD: Nonresearch determination
PLHIV: People living with HIV
PR: Prevalence ratio
RBC: Rwanda biomedical center
RNEC: Rwanda National Ethics Committee
RPHIA: Rwanda Population–Based HIV Impact Assessment
SSA: Sub‐Saharan Africa
UNAIDS: Joint United Nations Programme on HIV/AIDS
VIF: Variance inflation factor
VL: Viral load
VLS: Viral load suppression
WHO: World Health Organization

## Author Contributions

G.N.R.: conceptualization, methodology, original manuscript writing. J.C.K.: conceptualization, original manuscript review and editing. S.K.: methodology, data analysis. T.D.: writing–review and editing. C.H.: original manuscript review and editing. A.A.: original manuscript review and editing. A.T.: original manuscript review and editing. E.R.: original manuscript review and editing. D.H.P.: supervision, original manuscript review and editing. T.R.G.: supervision, original manuscript review and editing. All authors reviewed the manuscript and approved the final version for submission.

## Funding

This study received no specific funding.

## Ethics Statement

Rwanda’s CBS system was launched jointly by the Rwanda Biomedical Centre (RBC) and the U.S. CDC. It was approved in 2018 (tracking number 2018–309) and subsequently reviewed by the Rwanda National Ethics Committee (approval No. 907/RNEC/2019, November 18, 2019), which approved verbal, rather than written, informed consent for active CBS, recency testing, and routine follow‐up, in line with national HIV treatment program procedures. CBS was granted an NRD in 2021 by RBC and CDC, after which it became part of Rwanda’s routine surveillance system. All data used in this secondary analysis were generated through this programmatic national surveillance system, without personal identifiers being shared with third parties, and access to the de‐identified CBS dataset was administratively authorized by RBC under its institutional data‐sharing governance procedures.

## Conflicts of Interest

The authors declare no conflicts of interest.

## Supporting Information

Additional supporting information can be found online in the Supporting Information section.

## Supporting information


**Supporting Information** Table S1. Baseline characteristics of AYALHIV included in the analytic cohort compared with those excluded due to missing viral load results. Table S2. Variance inflation factors (VIF) for the multivariable model. Table S3. Test of the prespecified baseline CD4 × WHO clinical stage interaction. Table S4. Facility‐level clustering assessment (intraclass correlation coefficient). Table S5. Model goodness‐of‐fit and discrimination statistics. Table S6. Assessment of influential observations (Cook’s distance)

## Data Availability

The main data supporting the findings of this study are contained within the manuscript. The dataset used may be made available from RBC upon reasonable request and subject to RBC’s institutional approval and data‐sharing governance procedures.
