# Supplementary material for: Factors Associated With Viral Load Suppression Among Adolescents and Young Adults Living With HIV on ART in Rwanda: Data From Rwanda HIV Case‐Based Surveillance, 2019–2025
Source: J Trop Med. 2026 Aug 25;2026:5304043. doi: 10.1155/jotm/5304043 (PMC13504361; doi:10.1155/jotm/5304043)
Supplement: Supplementary file 1 [file JOTM-2026-5304043-s001.docx]

# Supplementary Tables

***Manuscript: “Factors associated with viral load suppression among adolescents and young adults living with HIV on ART in Rwanda: Data from Rwanda HIV Case-based surveillance, 2019-2025”***

Table S1. Baseline characteristics of AYALHIV included in the analytic cohort compared with those excluded due to missing viral load results

| **Characteristic** | **Included in analysis, N=4,140** | **Excluded, no VL recorded, N=494** |
| --- | --- | --- |
| **Sex: Female** | 3,633 (88%) | 432 (87%) |
| **Sex: Male** | 507 (12%) | 62 (13%) |
| **Age (years), median (Q1, Q3)** | 23 (21, 24) | 22 (20, 23) |
| **Age group: 15-19** | 386 (9.3%) | 78 (16%) |
| **Age group: 20-24** | 3,754 (91%) | 416 (84%) |
| **Marital status: Single** | 2,493 (60%) | 296 (61%) |
| **Marital status: Married/cohabitant** | 1,471 (36%) | 167 (34%) |
| **Marital status: Divorced/Separated** | 169 (4.1%) | 25 (5.1%) |
| **Baseline CD4: <200 cells/mm3** | 297 (7.9%) | 46 (12%) |
| **Baseline CD4: 200-499 cells/mm3** | 1,664 (44%) | 169 (44%) |
| **Baseline CD4: >=500 cells/mm3** | 1,816 (48%) | 173 (45%) |
| **WHO stage: I/II** | 4,088 (99%) | 475 (98%)* |
| **WHO stage: III/IV** | 50 (1.2%) | 10 (2.1%)* |
| **Initial regimen: DTG-based** | 3,774 (92%) | 449 (93%) |
| **Initial regimen: Non-DTG-based** | 344 (8.4%) | 35 (7.2%) |

*ART = Antiretroviral Therapy; CD4 = CD4+ T-Cell Count; DTG = Dolutegravir; VL = Viral Load; WHO = World Health Organization*

Table S2. Variance inflation factors (VIF) for the multivariable model

| **Variable** | **GVIF** | **Df** | **GVIF^(1/(2*Df))** |
| --- | --- | --- | --- |
| **Sex** | 1.020025 | 1 | 1.009963 |
| **Age group** | 1.012276 | 1 | 1.006119 |
| **Marital status** | 1.020718 | 2 | 1.005140 |
| **Baseline CD4** | 1.016546 | 2 | 1.004111 |
| **WHO stage** | 1.015745 | 1 | 1.007842 |
| **Initial regimen** | 1.087536 | 1 | 1.042850 |
| **Time to VL test** | 1.106876 | 2 | 1.025710 |
| **Adherence** | 1.002994 | 1 | 1.001496 |

*GVIF^(1/(2*Df)) squared is comparable to a standard VIF; values below 5 indicate acceptable multicollinearity.*

*CD4 = CD4+ T-Cell Count; Df = degrees of freedom; GVIF = Generalized Variance Inflation Factor; VIF = Variance Inflation Factor; WHO = World Health Organization*

Table S3. Test of the pre-specified baseline CD4 x WHO clinical stage interaction

| **Test** | **F statistic** | **Numerator df** | **Denominator df** | **p-value** | **Conclusion** |
| --- | --- | --- | --- | --- | --- |
| Baseline CD4 x WHO stage | 0.558 | 2 | 3,633 | 0.572 | Not significant: interaction excluded from the final model |

Table S4. Facility-level clustering assessment (intraclass correlation coefficient)

| **Metric** | **Value** |
| --- | --- |
| **Observations in model** | 3,645 |
| **Facility clusters** | 458 |
| **Adjusted ICC** | 0.016 |
| **Unadjusted ICC** | 0.016 |

*ICC = Intraclass Correlation Coefficient.*

Table S5. Model goodness-of-fit and discrimination statistics

| **Statistic** | **Value** |
| --- | --- |
| **Hosmer-Lemeshow chi-squared** | 3.313 |
| **Hosmer-Lemeshow df** | 3 |
| **Hosmer-Lemeshow p-value** | 0.346 |
| **Tjur's R2** | 0.044 |
| **Brier score** | 0.028 |

*df = degrees of freedom; R2 = R-squared (coefficient of determination).*

Table S6. Assessment of influential observations (Cook's distance)

| **Metric** | **Value** |
| --- | --- |
| **Method** | Cook's distance |
| **Observations flagged as influential** | None detected |

*Cook's distance flags observations with disproportionate influence on the fitted regression coefficients of the candidate*
